# CEA-targeted fluorescence endoscopy for the detection of high-grade dysplasia and early invasive rectal cancer: A proof-of-concept trial

**DOI:** 10.1007/s00259-026-08155-z

**Published:** 2026-09-03

**Authors:** M. I. Warmerdam, T. H. Dijkhuis, D. J. G. Linders, C. M. W. Pesch, A. S. L. P. Crobach, A. Matoshi, K. Basiliya, S. H. van den Berg, F. Cailler, K. C. M. J. Peeters, F. A. Holman, J. S. D. Mieog, A. L. Vahrmeijer, D. E. Hilling, J. J. Boonstra

**Affiliations:** 1 Department of Surgery, Leiden University Medical Center, Albinusdreef 2, 2333 ZA Leiden, The Netherlands; 2 Department of Pathology, Leiden University Medical Center, Albinusdreef 2, 2333 ZA Leiden, The Netherlands; 3 Department of Gastroenterology, Leiden University Medical Center, Albinusdreef 2, 2333 ZA Leiden, The Netherlands; 4 Surgimab SAS, 10, Parc Club du Millénaire, 1025 Avenue Henri Becquerel, 34000 Montpellier, France; 5 Department of Surgical Oncology and Gastrointestinal Surgery, Erasmus MC Cancer Institute, University Medical Center Rotterdam, Dr. Molewaterplein 40, 3015 GD Rotterdam, The Netherlands

**Keywords:** Fluorescence-guided endoscopy, Early colorectal cancer, Colorectal polyps, CEA-targeted imaging

## Abstract

**Purpose:**

Accurate recognition of high-grade dysplasia (HGD) and early invasive carcinoma (IC) within colorectal polyps is essential for selecting the preferred local resection technique. However, optical endoscopic diagnosis can be challenging, particularly in large and advanced polyps. This study investigates whether carcinoembryonic antigen (CEA)-targeted near-infrared fluorescence (NIRF) imaging can distinguish polyps containing HGD/IC from polyps solely containing low-grade dysplasia (LGD), given prior evidence of CEA overexpression in HGD/IC relative to LGD.

**Methods:**

Patients scheduled for local en-bloc endoscopic resection of advanced rectal polyps were included in the study and received 5 mg or 10 mg intravenous SGM-101, a CEA-targeted fluorescent agent. In vivo imaging was performed using a rigid Quest-Olympus NIRF-endoscope, followed by ex vivo whole-specimen and bread-loaf imaging using the PEARL imaging system. The primary endpoint was the NIRF tumour-to-low-grade-dysplasia ratio (TDR; HGD/IC divided by LGD) performed on bread loafs, as this method enabled direct pathological correlation. Secondary endpoints included dose-finding as well as sensitivity and specificity for detecting HGD/IC.

**Results:**

A total of 22 patients were included, with 9/22 containing LGD only and 13/22 containing HGD/IC. Eleven patients received 10 mg and 11 received 5 mg SGM-101. Ex vivo bread-loaf imaging showed a median (IQR) TDR of 1.7 (1.3–2.9), with higher values in the 10 mg group of 2.5 (2.0-3.5) compared to the 1.3 (1.2–1.6) in the 5 mg group (*p* = 0.023). The sensitivity for detecting HGD/IC was 86% (2 false-negatives), and specificity was 88% (1 false-positive).

**Conclusion:**

Although with limited sample size, this study demonstrates proof-of-concept of CEA-targeted NIRF-imaging in differentiating HGD/IC bearing polyps from completely benign LGD polyps. Clinical utility, including its impact on treatment decision-making and patient outcomes, remains to be demonstrated in future, larger prospective studies.

**Clinical trial registration number:**

NCT06280690.

**Supplementary Information:**

The online version contains supplementary material available at https://doi.org/10.1007/s00259-026-08155-z.

## Introduction

Population-based screening programs have led to the detection of a growing number of large colorectal polyps. In the Netherlands alone, approximately 4000 patients are diagnosed with large advanced adenomas (> 2 cm) or early stage rectal cancer [1, 2]. The type of resection warranted for these polyps depends on the polyp’s characteristics [3]. Benign polyps, solely existing of low-grade dysplasia (LGD) can be removed using piecemeal endoscopic techniques, a widely practiced procedure accessible to every endoscopist and hospital. In contrast, polyps containing high-grade dysplasia (HGD) or early invasive carcinoma (IC) require local en-bloc resection techniques, such as endoscopic submucosal dissection (ESD) or endoscopic intermuscular dissection (EID) [3]. These methods are more complex, costly, and require specialized training limited to a subset of endoscopists and hospitals [4].

Currently, treatment stratification for patients with rectal polyps relies on endoscopic optical diagnosis, endorectal ultrasound, and/or MRI. However, these imaging modalities have limitations in accurately distinguishing entirely benign LGD polyps from polyps containing HGD or early invasive cancerous areas and in assessing the depth of invasion [4–8].

Misclassification of colorectal lesions has significant consequences. Understaging HGD/IC-bearing polyps as LGD-polyps can result in inappropriate piecemeal rather than en-bloc resection, increasing the risk of uncertain margin assessment, incomplete (R1) resections and compromised evaluation of lymph node metastasis risk factors - all potentially necessitating additional major abdominal surgery, having substantial complication risks [3, 4, 9]. Among experts, 13%−22% of HGD/IC bearing polyps go unrecognized, even rising to as high as 81% in community settings [4, 6, 7]. Conversely, overstaging benign polyps as malignant can prompt unnecessary referrals for en-bloc resection. Due to the limited accuracy, current Dutch guidelines recommend that for patients with rectal polyps larger than 2 cm en-bloc resection should be considered. Unfortunately, only ~ 10% of these polyps turn out to contain a malignant component [4, 10]. Similarly, a large U.S. study showed that 25% of patients undergoing major abdominal surgery for a suspected malignant polyp had benign disease [11].

Therefore, there is a need for innovative imaging techniques to enhance polyp assessment. Tumour-targeted fluorescence-guided surgery (FGS) has emerged as a promising technique that enables real-time visualization of lesions based on specific molecular features rather than morphology [12, 13]. To apply FGS, the patient must be administered a tumour-targeted near-infrared fluorescent (NIRF) agent that specifically binds to an overexpressed protein on malignant cells [12, 13]. Additionally, a camera system is required to excite the fluorophore and visualize it in real-time on a screen [12, 13]. A recent study demonstrated that carcinoembryonic antigen (CEA) is a potentially reliable marker for distinguishing HGD and early IC from LGD and mucosa, as CEA was overexpressed in approximately 75% of HGD/IC cases, while nearly absent in LGD and mucosa in respectively 66% and 98% of cases [14]. CEA can be targeted using the fluorescent agent SGM-101, a chimeric anti-CEA antibody conjugated to the fluorophore BM-105. This agent has shown the ability to accurately visualize CEA-positive colorectal and pancreatic adenocarcinomas in multiple trials and is currently being evaluated in a phase III clinical trial for patients with locally advanced and metastasized colorectal cancer (NCT03659448).

This study aimed to evaluate the ability of SGM-101 to differentiate between various stages within a polyp, specifically HGD/IC, LGD, and normal mucosa. To achieve this, a cohort of patients scheduled for endoscopic en-bloc resection of advanced rectal polyps were infused with SGM-101, followed by in vivo and ex vivo fluorescence assessments.

## Materials and methods

### Trial design, inclusion criteria and sample size

This phase II, prospective, open-label clinical trial was conducted at the Leiden University Medical Center, a tertiary referral centre for local endoscopic resection of advanced rectal polyps in the Netherlands. Patients scheduled for local endoscopic en-bloc resection of an advanced rectal polyp, were eligible. Additionally, lesions had to be non-pedunculated, > 2 cm, suspected of HGD or superficial/deep submucosal invasion, and located within 15 cm from the anal verge on endoscopy. Endoscopic images from referral centres were reviewed by two endoscopists (JB, KB), requiring at least one white light (WL) and one narrow band (NBI) image. Based on these images, polyps were classified using the Kudo and Hiroshima criteria. If uncertain, a staging rectoscopy was performed. For Kudo Vn or Hiroshima C3 lesions, an MRI was performed to exclude patients with suspicious lymph nodes or advanced T2 or T3 tumours. Exclusion criteria included prior administration of SGM-101, pregnancy or breastfeeding, a history of anaphylactic shock and those who had any condition that the investigator considered to be potentially compromising to the patients’ wellbeing or the study objectives. The study was approved by the Medical Ethics Review Committee Leiden-Den Haag-Delft (METC LDD), Leiden, The Netherlands, on 23 August 2023 (Reference study number P23.020). The study was registered at ClinicalTrials.gov (NCT06280690). The study was conducted in accordance with the Declaration of Helsinki, Good Clinical Practice (ICH-GCP) guidelines, and applicable Dutch laws and regulations governing medical research involving human subjects. Written informed consent was obtained from all participants before enrolment in the study. Written informed consent for publication of anonymized data was obtained from all participants. All authors had access to the study data and reviewed and approved the final manuscript.

The study aimed to include 20 patients; based on a historical cohort where 70% of ESD specimens contained IC [15], this would result in approximately 15 cases in which fluorescence of the HGD/IC regions could be assessed, alongside about five negative controls with only LGD, a distribution considered sufficient for initial proof-of-concept testing. If a patient could not be analysed for the main endpoint (bread-loaf analysis), an additional patient would be enrolled.

### Study procedures

**Fig. 1:**
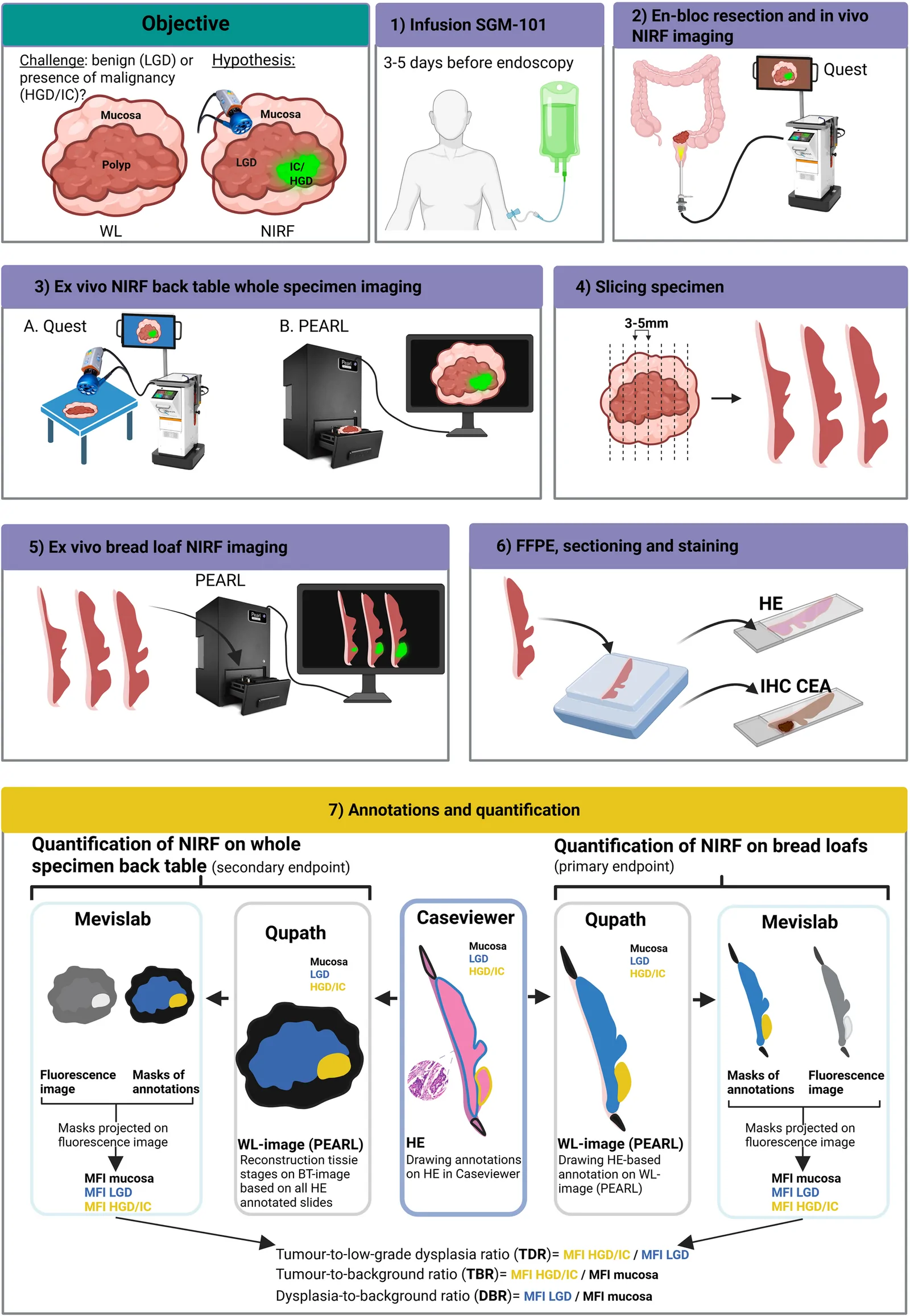
Overview of study workflow and NIRF imaging analysis. Patients scheduled for en-bloc resection of a rectal polyp were recruited for the study, after informed consent the following study procedures were performed: (1) Patients received an intravenous infusion of SGM-101 3–5 days prior to surgery; (2) before endoscopic en-bloc resection was performed, in vivo NIR fluorescence (NIRF) imaging using a rigid laparoscope (Quest-Olympus) was used for fluorescence assessment; (3) Ex vivo imaging of whole resected specimens was conducted on the back table using Quest and PEARL systems; (4) Specimens were sliced into 3–5 mm bread loaf sections; (5) Bread loaf sections were imaged with the PEARL system for NIRF analysis; (6) After formalin fixation and paraffin embedding (FFPE), tissue sections were stained with haematoxylin and eosin (H&E) and CEA immunohistochemistry; (7) Annotations were made with the pathologist on the H&E slides, and quantification of fluorescence intensities was performed using QuPath (annotations on white light PEARL images based on H&E), then exported to MeVisLab software for calculation of MFI and ratios including tumour-to-low-grade-dysplasia ratio (TDR), tumour-to-background ratio (TBR), and low-grade-dysplasia-to-background ratio (DBR). This was done for both bread loaf sections (primary endpoint) and whole specimens. *CEA* carcinoembryonic antigen, *FFPE* formalin-fixed paraffin-embedded, *HGD* high-grade dysplasia, *HE* haematoxylin and eosin staining, *IHC* immunohistochemistry, *LGD* low-grade dysplasia, *MFI* mean fluorescence intensity, *NIRF* near-infrared fluorescence, *WL* white light

Patients were admitted approximately four days (± 1 day) prior to endoscopic resection for administration of SGM-101. The drug was administered via a 30-minute intravenous infusion, followed by a 50 mL saline flush (0.9% sodium chloride) (Fig. 1, image 1). Patients were monitored for two hours post-infusion for potential (serious) adverse events ((S)AEs). SGM-101 was supplied by Surgimab (Montpellier, France).

As was described in the protocol, the first three patients received 10 mg of SGM-101, based on the optimal dose established in prior studies of advanced colorectal cancer patients undergoing major colorectal surgery [16]. Anticipating that a lower dose would be adequate thanks to the direct, close-range visualization during intraluminal endoscopy, the next three patients received 5 mg. An interim-analysis was conducted after these six patients to decide whether to proceed with 5 mg or 10 mg. As the interim-analysis was not able to conclusively establish the superiority of either dose in differentiating HGD/T1 from LGD, it was decided that the remaining patients were evenly assigned, with half of the study patients receiving 5 mg and half receiving 10 mg, completing the 5 mg group first before proceeding with the 10 mg group.

All endoscopic procedures were performed by experienced gastroenterologists specialized in endoscopic en-bloc resections of advanced polyps. After propofol sedation by the anaesthesiologist, patients underwent an initial routine endoscopic (non-fluorescence) inspection using standard flexible endoscopy equipment using an Olympus GIF-EZ1500 or GIF-HQ190. During the inspection, narrow band imaging (NBI) and near focus were used to identify areas suspected of submucosal invasion. Additionally, the polyp’s morphology was described based on the Paris classification, granularity, Kudo pit pattern, and Hiroshima type. As the Olympus flexible endoscope is not currently equipped with NIRF-technology, the Quest Spectrum 2.0/3.0 rigid laparoscope (Quest-Olympus, Wieringerwerf, The Netherlands) was utilized for fluorescence imaging assessment (Fig. 1 – image 2). The laparoscope was introduced transanally through a 12 × 100 mm balloon trocar (Kii^®^ Balloon Blunt Tip System, Applied Medical, Rancha Santa Margarita, USA). Working space was created through CO2-insufflation. Images of the polyp were captured, and the presence of fluorescent signals was noted.

Based on endoscopic imaging at time of the procedure the dissection plane was chosen by the endoscopist. In case of HGD or superficial invasion the submucosal plane was selected (ESD). In case of deep submucosal invasion an intermuscular dissection plane was used (EID). In case of a an even deeper rectal lesion an endoscopic full thickness resection was optional (eFTR). Following resection, the specimen was fixated with pins, and imaged ex vivo on the back table using the Quest Spectrum open camera 2.0/3.0 and the PEARL MSI imaging system (Li-Cor, Lincoln, USA) (Fig. 1, image 3), a close-field system that allows for standardized and reproducible imaging analysis. Fluorescent signals detected by both the PEARL and QUEST system (in vivo and ex vivo) were marked and correlated with pathological findings.

At the department of Pathology, the lesion was sectioned into bread loafs of 3–5 mm (Fig. 1, image 4). Each bread loaf was imaged with the PEARL (Fig. 1, image 5, main endpoint analysis) and processed into a formalin-fixed, paraffin-embedded (FFPE) block. From each FFPE block, two consecutive 4 μm sections were mounted on adhesive slides (Fig. 1, image 6); one was stained with H&E, the other used for CEA immunohistochemistry (IHC). In collaboration with a gastrointestinal pathologist, annotations of the mucosa, LGD, HGD and invasive carcinoma were made on a minimum of three representative H&E slides. These annotations were then used to quantify mean fluorescence intensity (MFI) for each stage within the fluorescent PEARL image of the bread loaf (see section ‘NIR fluorescence image analysis’) and to reconstruct all stages on the back table image (Fig. 1, image 7). CEA-expression was scored separately for each polyp stage on three tissue sections where possible, in consultation with the pathologist. Mean MFI and CEA-score were calculated per patient.

### Endpoints

The feasibility of SGM-101 in distinguishing HGD/IC from LGD or mucosa can most accurately be assessed using bread-loaf specimens, as these allow direct correlation with H&E-stained histological slides. Therefore, the primary outcome of this study was the ex vivo bread loaf fluorescence tumour-to-low-grade-dysplasia ratio, TDR (HGD/IC to LGD ratio) (Fig. 1, image 7, right illustration). Secondary outcomes were the tumour-to-background ratio, TBR (HGC/IC to mucosa ratio) and the low-grade-dysplasia-to-background ratio, DBR (LGD to mucosa ratio) on bread loafs.

Other secondary endpoints consisted of the TDR, TBR and DBR on ex vivo whole specimen imaging on the back table (Fig. 1, image 7, left illustration). Another secondary endpoint was the accuracy of SGM-101 to accurately identify HGD/IC in vivo and ex vivo on the back table. A polyp containing HGD/IC that exhibited fluorescence at the site of malignancy was considered a true positive (TP), while in the absence of fluorescence at the site of malignancy was classified as a false negative (FN). A polyp that did not contain HGD/IC but showed fluorescence was considered a false positive (FP), while in the absence of fluorescence was classified as a true negative (TN). Sensitivity, specificity, positive predictive value (PPV) and negative predictive value (NPV) were assessed. Endoscopist accuracy in detecting HGD/IC during initial WL inspection was recorded. Polyps with Kudo Vn/Vi or Hiroshima C1–C3 features were classified as HGD/IC. Other endpoints were the optimal dose to highlight HGD/IC with the highest contrast to LGD and mucosa while maintaining a low DBR, the relation between CEA-expression per stage of the polyp and MFI and the relation between MFI of the HGD/IC and deepness of invasion. The depth of invasion was stratified into superficial (HGD and T1SM1) and deep (T1SM2, T1SM3, and T2) categories, each requiring distinct treatment approaches: ESD for superficial and EID for deep invasion.

At the end of the trial a flexible fibre NIRF-endoscope came available (Olympus Fiber endoscope, CF-E3L, Insertion tube diameter 12.9 mm, field of view 140°, depth of field 5–100 mm) and was tested in patients 21 and 22 ex vivo and optically compared to the rigid laparoscope.

### NIRF image analysis

The semi-automatic standardized NIRF-analysis method used in this study has been previously described in detail by Dijkhuis et al., and has demonstrated effectiveness, objectivity, and reproducibility [17]. In collaboration with a gastrointestinal pathologist (SC), annotations of mucosa, LGD and HGD/IC were made in Caseviewer (v2.9) on at least three representative H&E slides. LGD was defined by mild to moderate architectural and cytological abnormalities confined to the superficial epithelium, with well-formed glands and limited nuclear atypia; HGD was characterized by severe architectural distortion and cytological atypia throughout the epithelium, with marked nuclear pleomorphism and increased mitotic activity. IC was defined by cancer cells breaching the basal lamina and invading the underlying stroma or muscle layers, indicating a transition from dysplasia to invasive malignancy. The pathologist was blinded to the fluorescence results. Next, the bread-loaf brightfield and NIRF-images were imported into QuPath (V0.3.2), an open-source image analysis software. Based on the H&E annotations, identical annotations were manually applied to the macroscopic WL PEARL image to delineate mucosa, LGD, and HGD/IC (Fig. 1, image 7, right panel). Annotations were performed on the WL image rather than the fluorescence image to prevent selection bias, as the operators are not influenced by the fluorescent signal. Subsequently, these PEARL annotations and the identical NIRF-image were imported into MeVisLab (V3.4.1), a medical image processing framework. Annotation masks were projected onto the NIRF-image, and MFI values for each annotation mask was extracted. These MFIs were then used to calculate the TDR, TBR, and DBR. Back table whole specimen images were analysed similarly; however, since direct correlation with H&E staining was not possible, annotations (mucosa, LGD and HGC/IC) were reconstructed based on the H&E annotations on tissue slices (Fig. 1, image 7, left panel).

### Immunohistochemistry and scoring

CEA expression for each polyp stage was assessed by IHC using a monoclonal mouse antibody against CEACAM5 (clone CI-P83-1, Santa Cruz) at 1:1000 dilution, following heat-induced antigen retrieval at 95 °C for 10 min in PT Link (Dako) with low pH (6.0). A dedicated pathologist scored each stage using the IRS method, multiplying staining intensity (0 = absence, 1 = weak, 2 = moderate, 3 = strong) by the percentage of positive cells (1 = 25%, 2 = 50%, 3 = 75%, 4 = 100%). Due to observed heterogeneity, a pre-validated modification was applied [18]. This adjustment involved scoring each intensity separately. For example, if a specimen contained 50% tumour cells with moderate intensity (2 × 2 = 4) and 50% with strong intensity (2 × 3 = 6), the total score was 10. The pathologist was blinded to the fluorescence results.

### Statistical analysis

Statistical analyses were performed using IBM SPSS Statistics V25 (IBM Corp., Armonk, USA) and GraphPad Prism V10.2.3 (GraphPad Software Inc., La Jolla, USA). Figures were made using GraphPad Prism and Biorender. Descriptive statistics were used to summarize patient clinical characteristics. Baseline characteristics were compared using the Fisher’s exact test for categorical variables. For unpaired data, the unpaired t-test was used for normally distributed continuous variables, and the Mann–Whitney U test for ordinal or non-normal data. For paired data, the paired t-test was applied to normally distributed variables, and the Wilcoxon signed-rank test for ordinal or nonparametric data.

## Results

### Study flow and cohort characteristics

**Fig. 2:**
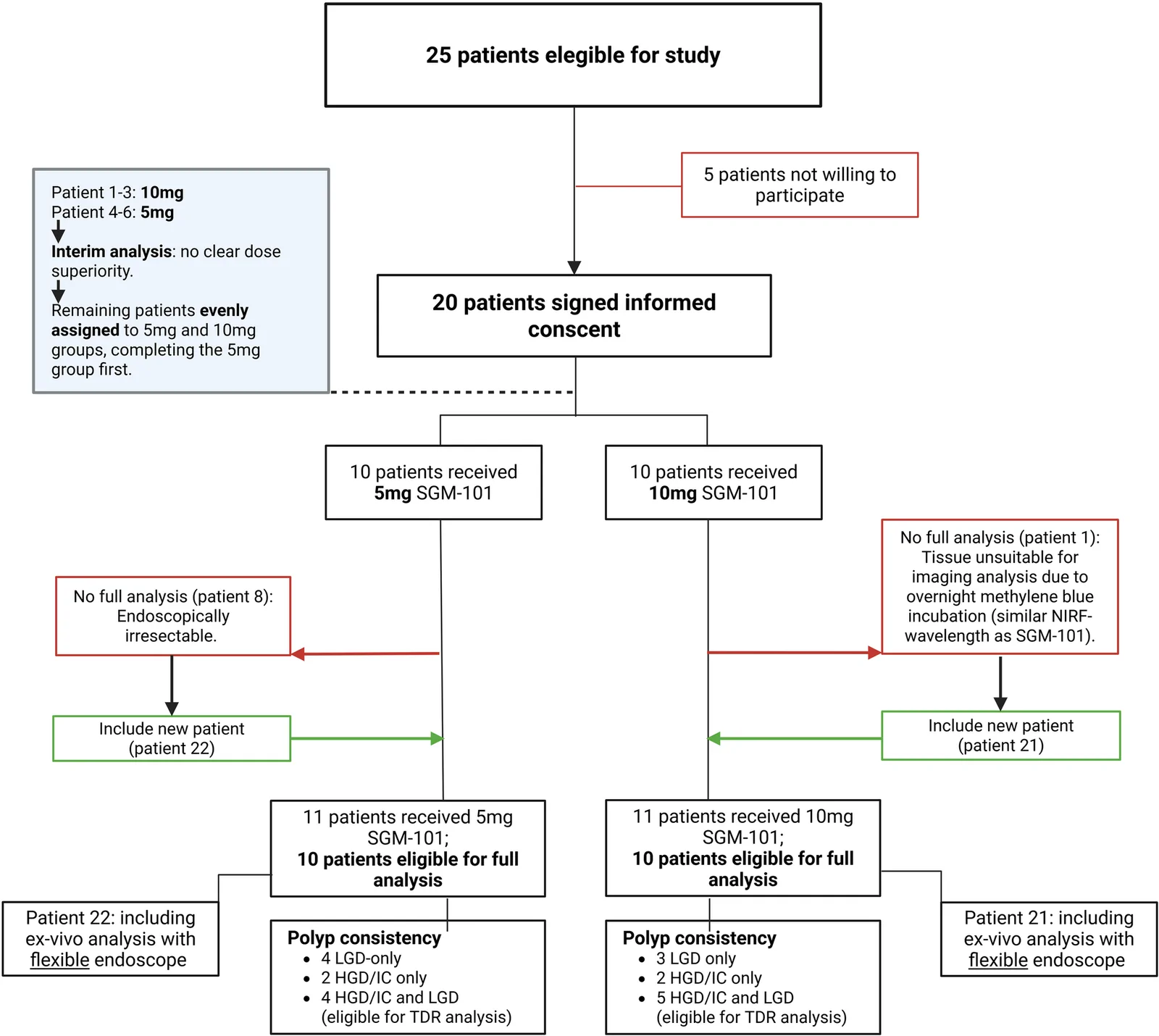
Patient flowchart showing enrolment, treatment, and polyp pathology. In total, 22 patients were evenly assigned to 5 mg (*n* = 11) or 10 mg (*n* = 11) SGM-101. Full analysis on all endpoints included 10 patients per group. Patient 21 and 22 underwent ex-vivo imaging with a NIRF flexible endoscope (Quest-Olympus). *HGD* high-grade dysplasia, *IC* invasive carcinoma, *LGD* low-grade dysplasia, *NIRF* near-infrared fluorescence, *TDR* tumour-to-low-grade-dysplasia ratio

The patient flow is shown in Fig. 2. The study aimed to enrol 20 patients. Unfortunately, two enrolled participants were not evaluable for the primary outcome. Consequently, two additional patients were included. Patients were evenly split between 5mg and 10mg groups (n=11 each). Patients 21 and 22 underwent an additional ex vivo imaging assessment with the Quest-Olympus flexible fibre endoscope, which became available at that time. In total, 13 polyps (65%, of 20 fully evaluable) were classified HGD/IC as maximal degree of disease. Of these, 4 polyps (20%) contained HGD/IC only (no LGD; not available for TDR-analysis), and 9 (45%) contained both HGD/IC and LGD (eligible for TDR analysis). Among the latter, 4 were in the 5mg group and 5 in the 10mg group.

All baseline characteristics are summarized in Table 1. The mean (+SD) age was 66 + 10 years and 17 (77%) patients were male. Ten patients (44%) underwent ESD, nine (41%) underwent EID, two (9%) underwent eFTR, and one polyp was irresectable (5%).

**Table 1:** Baseline characteristics, polyp features, and pathology of patients receiving 5 mg or 10 mg SGM-101

	5 mg (*n* = 11)	10 mg (*n* = 11)	Total (*n* = 22)	*p*-value
Patient characteristics				
Male, n (%)	8 (73)	9 (82)	17 (77)	0,5
Age in years, mean (SD)	66 (9)	65 (11)	66 (10)	0,816
BMI, median (IQR)	27 (25–28)	24 (23–26)	25 (24–28)	0,047
CEA in blood, ug/ml, med (IQR)	3 (2–4)	2 (1–3)	2 (1–4)	0,293
Type of endoscopic resection, n (%)				0,494
ESD	6 (55)	4 (36)	10 (44)	
EID	3 (27)	6 (54)	9 (41)	
eFTR	1 (9)	1 (9)	2 (9)	
Irresectable	1 (9)	0 (0)	1 (5)	
Polyp characteristics endoscopy				
Distance from anal verge in cm, mean (SD)	8 (4)	8 (6)	8 (5)	0,895
Diameter polyp in mm, mean (SD)	40 (17)	29 (16)	35 (17)	0,096
Gross morphology				0,58
Sessile, n (%)	6 (55)	5 (45)	11 (50)	
Flat elevated, n (%)	0 (0)	1 (10)	1 (5)	
LST, n (%)	5 (45)	5 (45)	10 (45)	
Paris classification, n (%)				0,459
0-Is	3 (27)	2 (18)	5 (22)	
0-Is + C	0 (0)	2 (18)	2 (9)	
0-IIa + C	1 (9)	2 (18)	3 (14)	
0-IIa+excavated	1 (9)	0 (0)	1 (5)	
0-IIa + Is	6 (55)	5 (46)	11 (50)	
Granularity, n (%)				0,635
Granular	4 (36)	2 (18)	6 (27)	
Non-granular	7 (64)	9 (82)	16 (73)	
Kudo classification, n (%)				0,385
IIIL	2 (18)	0 (0)	2 (9)	
IV	2 (18)	2 (18)	4(18)	
Vi	3 (27)	4 (36)	7 (32)	
Vn	4 (37)	5 (46)	9 (41)	
Hiroshima, n (%)				0,350
B	4 (36)	12(18)	6 (27)	
C1	4 (36)	4 (36)	8 (36)	
C2	0 (0)	1 (10)	1 (5)	
C3	3 (28)	4 (36)	7 (32)	
Suspect for HGD/invasive carcinoma, n (%) *	8 (73)	9 (82)	17 (77)	1,00
Pathology **	*n* = 10	*n* = 10		
Maximal degree of disease, n (%)				0,292
LGD	4 (40)	3 (30)	7 (35)	
HGD/invasive carcinoma	6 (60)	7 (70)	13 (65)	
HGD	2 (20)	0 (0)	2 (10)	
T1SM1	1 (10)	2 (20)	3 (15)	
T1SM2	0 (0)	0 (0)	0 (0)	
T1SM3	2 (20)	2 (20)	4 (20)	
T2	1 (10)	3 (30)	4 (20)	
Main endpoint analysis, n(%)				
TDR	4 (40)	5 (50)	9 (45)	
TBR	6 (60)	7 (70)	13 (65)	
DBR	8 (80)	8 (80)	16 (80)	
Budding, bd2-3, n (%)	1 (10)	2 (20)	3 (15)	0,357
Lymphovascular invasion, n (%)	0 (0)	2 (20)	2 (10)	0,467
Perineural invasion, n (%)	1 (10)	0 (0)	1 (5)	0,476
CEA-expression, med (IQR)				
Mucosa	1 (1–1)	1 (1–2)	1 (1–1)	0,068
LGD	6 (6–7)	6 (5–6)	6 (6–6)	0,166
HGD/IC	9 (8–10)	10 (9–11)	10 (8–11)	0,490

Data are shown per dose group and total, with p-values for group comparisons

* If it was Kudo Vi/Vn or Hiroshima C1–C3, then it was classified as suspicious for HGD/IC

** Pathology data is shown for the patients that were fully evaluable on all endpoints (n=20)

*HGD* high-grade dysplasia, *IC* invasive carcinoma, *LGD* low-grade dysplasia, *BMI* body mass index, *IQR* interquartile range, *SD* standard deviation, *CEA* carcinoembryonic antigen, *ESD* endoscopic submucosal dissection, *EID* endoscopic intermuscular dissection, *EFTR* endoscopic full-thickness resection, *TDR* tumour-to-low-grade-dysplasia ratio, *TBR* tumour-to-background ratio, *DBR* low-grade-dysplasia-to-background ratio

The 5mg and 10mg groups did not differ in patient characteristics, type of endoscopic resection, polyp characteristics and pathology, except for a higher median BMI in the 5mg group of 27 (IQR 25-28) versus 24 (IQR 23-26) (p<0.047). More particularly, the two groups showed evenly maximal stage of disease and CEA-expression.

All 22 patients underwent fluorescent imaging in at least one stage of the protocol. Supplementary Fig. 1 shows in vivo and ex vivo WL and NIRF-images of three illustrative cases.

### Safety

In total, five adverse events (AEs) and two serious adverse event (SAE) were reported, none related to the drug or study-procedure (Supplementary table 1).

### Quantitative NIRF assessments

#### Ex vivo bread-loaf NIRF evaluation - main study endpoint

**Fig. 3:**
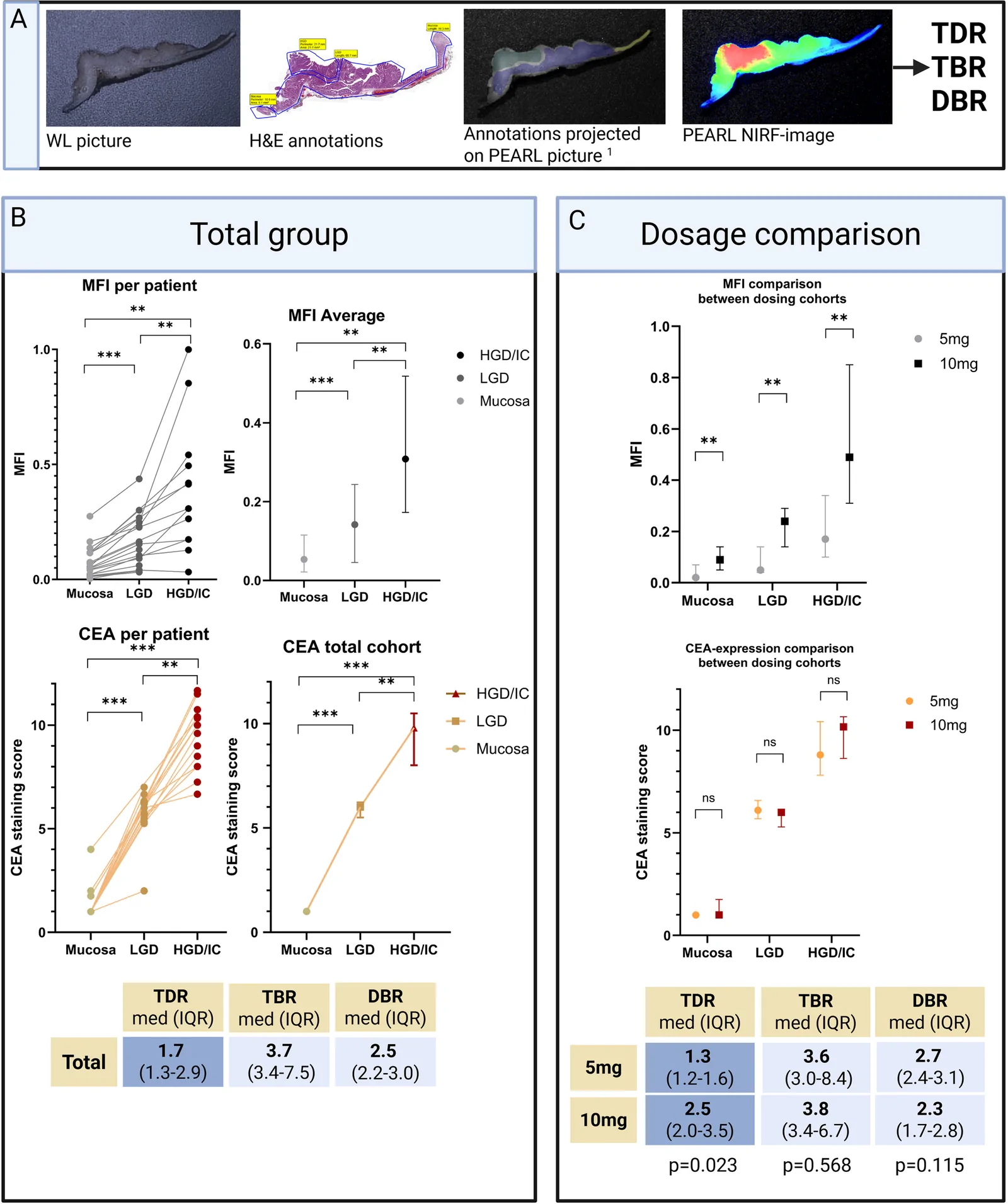
Ex-vivo fluorescence quantification and CEA scoring on bread loafs, main endpoint (**A**) Illustration of workflow of ex-vivo bread-loaf analysis, from left to right: white light picture of a bread-loaf specimen (patient 19). To the right the H&E-slice with annotations (mucosa, LGD, HGD/IC) by the pathologist. To the right the H&E annotations projected on the PEARL picture. To the right a fluorescence picture according to a heat-map projection. From the annotations on the fluorescence picture, MFIs are subtracted and subsequently TDR, TBR and DBR are calculated. (**B)** Total cohort analysis showing mean fluorescence intensity (MFI, top) and CEA staining scores (middle) increase significantly with histologic grade (mucosa < LGD < HGD/IC). Corresponding fluorescence ratios (TDR, TBR, DBR) are summarized below. (**C**) Dosage comparison reveals higher MFI in the 10 mg group, significantly so for HGD/IC, while CEA expression is similar between groups. TDR shows a trend favouring 10 mg (*p* = 0.06). *p < 0.05, **p < 0.01, ***p < 0.001, ns = not significant (*p* > 0.05). ^1^annotations: green = HGD/IC, blue = LGD, yellow=mucosa. *HGD* high-grade dysplasia, *IC* invasive carcinoma, *LGD* low-grade dysplasia, *MFI* mean fluorescence intensity, *CEA* carcinoembryonic antigen, *TDR* tumour-to-low-grade-dysplasia ratio, *TBR* tumour-to-background ratio, *DBR* low-grade-dysplasia-to-background ratio

As bread-loaf NIRF evaluation enables accurate sequential H&E-correlation, the primary endpoints were based on this method (Fig. 3a for illustration). In the overall cohort, median MFI values for mucosa, LGD, and HGD/IC were 0.05 (IQR 0.02-0.12), 0.14 (IQR 0.05-0.24), and 0.31 (IQR 0.17-0.52), respectively (Fig. 3b,upper illustrations), all three significantly different from each other (p<0.05). CEA increased correspondingly (Fig. 3b, center illustrations). The median TDR and TBR of 1.7 (IQR 1.3-2.9) and 3.7 (IQR 3.4-7.5) show good discrimination between HGD/IC versus LGD and mucosal tissue, respectively (Fig. 3b, lower illustration).

When comparing the two dosages within each tissue type (i.e., HGD/IC vs. HGD/IC, mucosa vs. mucosa and LGD vs. LGD), MFI values were significantly higher in the 10mg group (Fig. 3c, upper illustrations), while CEA expression was comparable between the dose cohorts (Fig. 3c, center illustrations). The 10mg group showed a higher TDR (2.5 vs. 1.3, p=0.023), while DBR was comparable (2.3 vs. 2.7, p=0.115, Fig. 3c, lower illustration).

#### Ex vivo whole specimen NIRF evaluation 

**Fig. 4:**
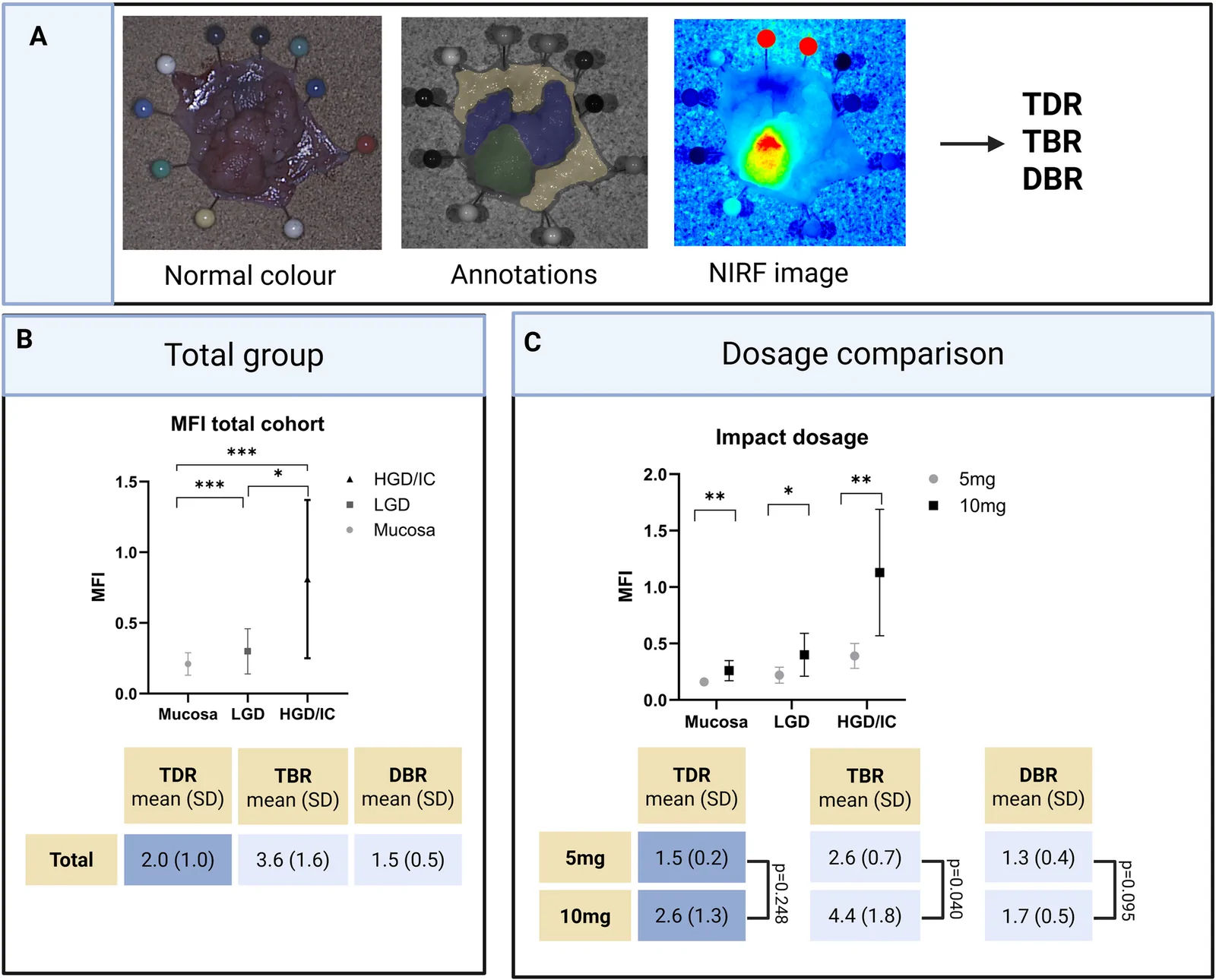
Ex-vivo whole specimen fluorescence tissue analysis distinguishes HGD/IC from LGD and mucosa (**A**) Illustration of workflow of ex-vivo whole specimen analysis with1 clinical image, H&E based reconstruction of annotations and fluorescence intensity picture. (**B**) Total cohort: MFI is lowest in mucosa, higher in LGD, and highest in HGD/IC. (**C**) Dosage comparison: 10 mg results in higher MFI than 5 mg in mucosa, LGD, and HGD/IC. All ratios are higher in 10 mg (*p* > 0.05). *p < 0.05, **p < 0.01, ***p < 0.001, ns = not significant (*p* > 0.05). ^1^annotations: green = HGD/IC, blue = LGD, yellow=mucosa. *WL* White light, *MFI* mean fluorescence intensity, *LGD* low-grade dysplasia, *HGD/IC* high-grade dysplasia/invasive carcinoma, *TDR* Tumour-to-low-grade-dysplasia ratio, *TBR* tumour-to-background ratio, *DBR* low-grade-dysplasia-to-background ratio

As a secondary endpoint, MFIs by tissue type (mucosa, LGD, HGD/IC) and corresponding fluorescence ratios were calculated ex vivo on whole specimen post-resection (Fig. 4a). Mean MFIs were 0.21 (SD 0.08) for mucosa, 0.30 (SD 0.16) for LGD, and 0.81 (SD 0.56) for HGD/IC, with all pairwise comparisons showing statistically significant differences (Fig. 4b). This corresponded to a mean TDR, TBR and DBR of 2.0 (SD 1.0), 3.6 (SD 1.6), and 1.5 (SD 0.5), respectively (Fig. 4b). The 10mg group demonstrated significantly higher MFIs in all three tissue types compared to the 5mg group (Fig. 4c). All three ratios were numerically higher in the 10mg group (Fig. 4c).

### Qualitative assessments

#### Intraluminal evaluation with routine WL endoscopy and NIRF

 Discrimination of LGD from HGD/IC polyps (kudo V or Hiroshima C) using routine WL endoscopic imaging yielded 14 TPs, 5 TNs, 2 FPs, and 1 FN, corresponding to a sensitivity of 93% (95% CI: 70–99%), specificity of 71% (95% CI: 36–92%), PPV of 88% (95% CI: 64–97%), NPV of 83% (95% CI: 44–97%), and an overall accuracy of 86% (95% CI: 67–95%) (Table 2, first row). In comparison, NIRF imaging yielded 11 TPs, 6 TNs, 1 FP, and 1 FN, resulting in a sensitivity of 92% (95% CI: 65–99%), specificity of 86% (95% CI: 49–97%), PPV of 92% (95% CI: 65–99%), NPV of 86% (95% CI: 49–97%), and an overall accuracy of 89% (95% CI: 69–97%) (Table 2, second row). NIRF imaging using the rigid laparoscope was not feasible in 3 of the 22 specimens due to limitations in the rigid endoscope’s angulation. Also, for the other 19 specimens, complete polyp visualization was occasionally suboptimal, highlighting the need for a flexible NIRF endoscope.

**Table 2:** Diagnostic performance of routine white-light endoscopy, in vivo fluorescence imaging, and ex-vivo analysis

	TP	FP	TN	FN	*N*.A.	Sensitivity (95% CI)	Specificity (95% CI)	PPV (95% CI)	NPV (95% CI)	Accuracy (95% CI)
WL Endoscopy	14	2	5	1	0	93%(70–99%)	71%(36–92%)	88%(64–97%)	83%(44–97%)	86%(67–95%)
In vivo (Quest)	11	1	6	1	3*	92% (65–99%)	86% (49–97%)	92% (65–99%)	86% (49–97%)	89% (69–97%)
Bt (PEARL)	14	4	4	0	0	100% (79–100%)	50% (22–78%)	78% (55–91%)	100% (51–100%)	82% (62–93%)
BT (Quest)	12	1	7	2	0	86% (60–96%)	88% (53–98%)	92% (67–99%)	78% (45–94%)	86% (67–95%)

*TP* true positive, *FP* false positive, *TN* true negative, *FN* false negative, *N.A.* not applicable, *PPV* positive predictive value, *NPV* negative predictive value, *WL* white-light, *BT* back table of whole specimen, *95% CI* 95% confidence interval (Wilson score method)

Patient 9 had both a true positive as a false positive fluorescent hotspot on the BT for Quest and PEARL

*For three lesions, the in-vivo rigid fluorescence scope was unable to bring the entire polyp into view because the correct camera angle could not be achieved

#### Ex vivo NIRF assessments

The sensitivity of the PEARL system for detecting HGD/IC was 100% (95% CI: 79–100%), higher than the 86% (95% CI: 60–96%) observed with the Quest Spectrum. Conversely, specificity was lower for the PEARL (50% - 95% CI: 22–78%) compared to the Quest (88% - 95% CI: 53–98%). Full diagnostic metrics are presented in Table 2, rows 3 and 4.

### Potential impact of SGM-101 as an adjunct on routine endoscopy for polyp-discrimination

Supplementary Table 2 presents diagnostic accuracy on a per-patient basis. Routine WL flexible endoscopy misclassified the polyp stage in 3 of 22 patients. Of these, NIRF imaging with the clinical Quest system correctly identified the stage in all three cases: Patients 2 and 13 (both LGD) were suspected of HGD/IC by endoscopy yet correctly lacked fluorescence. Patient 9 (HGD at pathology) was incorrectly identifed as LGD at WL-endoscopy while fluorescence correctly indicated fluorescence at the site of HGD.

Conversely, in three cases, routine WL endoscopy correctly assessed the polyp stage, while NIRF imaging with the clinical Quest system misclassified it, either in vivo or ex vivo. Patient 1 (pT2) showed no back-table fluorescence because the HGD/IC focus was obscured by LGD tissue, despite higher CEA levels in HGD/IC (9 vs. 6). Patient 6 (LGD) exhibited slight in vivo fluorescence at a site of LGD, even though CEA was not elevated. Patient 10 (T1SM1) lacked fluorescence despite a modest CEA increase in the HGD/IC area (8 vs. 6), likely insufficient for detection, as supported by a low TDR (1.3) in the bread-loaf analysis; notably, the more sensitive PEARL system was able to visualize it.

### Predictive value of TDR and TBR for depth of invasion

To assess whether TDR or TBR of HGD/IC could predict depth of invasion, lesions were categorized as superficial (HGD and T1SM1, n=7), most suitable for ESD-resection, or deep (T1SM2, T1SM3, and T2, n=9), most suitable for EID-resection. Median TDR and TBR were slightly higher in the deep invasion group; however, differences were not statistically significant in either the whole specimen or bread-loaf analyses (Supplementary fig. 2).

### Ex vivo flexible endoscopy imaging testing

Ex vivo flexible endoscopy using the Quest-Olympus flexible fibre endoscope was performed in patients 21 and 22 upon its availability. Fluorescence measurements obtained using the flexible and rigid laparoscopes appeared broadly comparable on visual inspection (Supplementary fig. 3), supporting its readiness for translation to in vivo application.

## Discussion

 In this initial proof-of-concept study, we show that SGM-101, a CEA-targeted fluorescent agent, is able to detect HGD/IC with strong accuracy and contrast, allowing effective differentiation from LGD and normal mucosa. Although this study was primarily conducted using a rigid laparoscope in-vivo, the first ex vivo results with the flexible endoscope were promising, supporting its readiness for future in-vivo translation.

 The primary endpoint of this study was the TDR measured on bread-loaf specimens, as this method offers the most accurate and reliable assessment. The observed median TDR was 1.7 (IQR 1.3-2.9), indicating reasonable contrast between HGD/IC and LGD. Notably, the 10mg group showed a higher median TDR of 2.5 (IQR 2.0–3.5), reflecting even more consistent and pronounced contrast in the majority of patients. The bread-loaf TBR of 3.7 indicates strong contrast between tumour tissue and mucosa. Unfortunately the DBR of 2.5 also suggests contrast between LGD and normal mucosa. However, both the TBR and DBR values were likely overestimated due to methodological limitations, as the mucosa’s small size and thin structure on bread loafs may have led to underestimation of its MFI, inflating TBR and DBR. This limitation was less evident in the ex vivo whole specimen analysis, where larger and more defined mucosal surfaces yielded TBR and DBR values of 3.4 and 1.5, respectively, likely better representing true fluorescence contrast. The relatively low NIRF contrast observed between LGD and normal mucosa in these measurements aligns with our clinical experience during NIRF evaluation, where LGD/adenomatous fluorescence signals were generally weak or absent while these in HGC/IC were often optically strong.

 The diagnostic accuracy of routine WL endoscopy observed in this study aligns with data from other expert tertiary care centres, where miss rates for early invasive carcinoma typically range between 13% and 22%. However, this stands in contrast to community-level settings, where miss rates have been reported to reach as high as 81% [4, 6, 7]. Therefore, routine WL endoscopic assessments results observed in this trial may not accurately reflect the performance of the average endoscopist in routine clinical practice. Consequently, the addition of fluorescence-guided endoscopy as an add-on to routine WL endoscopy could provide substantial benefits especially in the community setting by improving detection rates. Moreover, despite the already high accuracy of WL endoscopy in this trial -performed by an expert endoscopist- NIRF imaging using the clinical Quest system was still able to correctly reclassify the tumour stage in three cases (14%): two false positive assessments by routine endoscopy (judged HGD/IC, but LGD) correctly showed no NIRF signal, while one false negative assessment by routine endoscopy (judged LGD, but HGD) correctly demonstrated fluorescence at the HGD- focus. Unfortunately, in three other cases, NIRF assessments using the clinical Quest system, either in-vivo or ex vivo, were wrong (two false negative, one false positive), while routine endoscopic assessment was correct. Interestingly, for both false negatives, the PEARL system did show fluorescence at the site of malignancy, demonstrating its higher sensitivity compared to the clinical Quest system. However, this increased sensitivity came at the expense of more false positives.

 While acknowledging the limited group sizes, patients who received 10mg showed statistically higher MFI values within HGD/IC lesions, as well as higher TDRs compared to the 5mg group, while DBR remained similar. Given this trend and the visibly brighter signals with 10mg, we currently recommend 10mg of SGM-101 for intraluminal endoscopic imaging - consistent with the optimal dose previously identified for intra-abdominal tumour imaging [16, 19].

Another secondary objective was to assess whether the depth of invasion could be determined based on TDR/TBR. Invasive superficial lesions (HGD/T1SM1) can be resected using ESD, while invasive deep lesions require EID. Although the difference in TBR/TDR was not statistically significant, we did observe a trend toward higher TBR/TDR values in cases of deep invasion compared to superficial invasion. To better evaluate the potential of fluorescence in this context, a larger, adequately powered study is needed.

A recent study employed the non-specific fluorescent tracer indocyanine green (ICG) to discriminate between malignant and benign polyps [20]. ICG, a fluorescent dye that binds to intravasal albumin, functions as a tissue perfusion marker shortly after intravenous injection. The study demonstrated that malignant colorectal tumours exhibit lower ICG perfusion rates than normal mucosa and non-cancerous polyps [20]. Based on these results, the International multicentre CLASSICA trial (NCT05793554) has been initiated. Given that ICG is excited at approximately 800nm while SGM-101 at 680nm, these tracers are spectrally compatible and could potentially be used in combination. Two other landmark studies have also employed fluorescence-guided endoscopy, although with different objectives than our trial. Burggraaf et al. reported in Nature Medicine the successful identification of all dysplastic colonic lesions using a C-met targeted tracer, including nine additional polyps not visible under WL [21]. Tjalma et al. successfully assessed treatment response in neoadjuvant-treated rectal cancer patients using a fluorescent antibody targeting vascular endothelial growth factor A [22].

Despite the study’s strong methodology and design, some limitations exist. The pathological H&E based annotations for mucosa, LGD, HGD, and IC served as the gold standard in this study. However, polyp progression is a continuous process, and the transition from LGD to HGD may represent a grey zone, making precise demarcation challenging. To minimize ambiguity, transitional indistinct areas were excluded from annotation. A second limitation was the use of a rigid rather than a flexible endoscope, which restricted inclusion to distal rectal polyps and introduced technical challenges for proper visualization. In three patients, the polyp could not be visualized in-vivo, and in others, optimal positioning for imaging was difficult to achieve. However, as this was primarily a proof-of-concept of the imaging strategy rather than a validation of a deployable imaging platform, this was considered acceptable. Notably, a flexible endoscope was successfully tested ex vivo in the final two patients, supporting its suitability for future in vivo application. Despite the exploratory nature of this trial, the limited sample size remains an important limitation. The study included only 22 patients, of whom 13 had HGD/IC, and TDR calculation was possible in only 9 polyps. Therefore, the findings should be considered hypothesis-generating and warrant confirmation in larger validation studies. However, the inclusion of 9 patients without HGD/IC represents an important strength, as it allowed preliminary evaluation of specificity in addition to sensitivity. Another limitation is that CEA expression is heterogeneous across colorectal neoplasia. Although CEA is considered one of the most promising and extensively studied targets for colorectal cancer imaging, expression is not uniform across lesions; not all HGD/IC lesions demonstrate high CEA expression, and CEA expression may overlap between HGD/IC and LGD lesions [14, 23]. This biological heterogeneity likely contributes to false-negative findings and highlights the limitation of relying on CEA expression alone for clinical decision-making. Nevertheless, CEA remains a highly valuable target for colorectal cancer imaging, and CEA-targeted NIRF endoscopy represents a promising adjunct to conventional endoscopic assessment, as was illustrated by the results in this trial. A fifth overall limitation is that, although this technique has the potential to particularly benefit community-level screening endoscopists, widespread adoption in that setting remains a distant prospect. Successful implementation requires that all endoscopists have access to NIRF-compatible flexible endoscopes and the fluorescent agent, which are currently not widely available. Furthermore, the agent must be administered intravenously 3–5 days before the procedure, whereas ideally, the endoscopist should be able to decide during the examination whether to use NIRF for a complex polyp, without the need for prior infusion. A promising alternative is the topical application of fluorescent agents, which has gained attention in recent years [24]. Therefore, further research is needed to evaluate the feasibility of topical fluorescence administration for distinguishing malignant from benign lesions. Lastly it should be noted that predictive values are prevalence-dependent, the PPV/NPV reported in this trial cannot be directly extrapolated to lower-prevalence settings, like community-level screening endoscopists, where the prevalence of HGD/IC is substantially lower.

Fluorescence-guided surgery has significantly advanced, with a wide range of available fluorescent agents and commercial camera systems [12]. In contrast, fluorescence-guided endoscopy is still emerging, despite its vast potential. To date, no flexible endoscopes capable of detecting fluorescence have been commercialized. As a result, research teams have had to rely on in-house developed flexible endoscopes or rigid endoscopes. To accelerate the development of fluorescence-guided endoscopy, it is crucial that commercially available flexible fluorescence-detecting endoscopes reach the market. Our study marks an important step forward in this process by ex vivo testing a prototype flexible endoscope from one of the leading commercial camera manufacturers. Our results demonstrate a new clinical application for fluorescence-guided endoscopy, further strengthening the evidence for its broad potential, encouraging the development of flexible fluorescence-detecting endoscopes.

A future study building on this work should prioritize in vivo validation of the flexible fluorescence endoscope and further evaluate the agent's efficacy in distinguishing LGD-polyps from those containing HGD/IC areas in a larger cohort. Ultimately, the future study should assess the clinical utility and societal benefits of this approach. We envisage two possible pathways towards clinical implementation. First, ideally, CEA-targeted fluorescence endoscopy would be applied to all patients undergoing screening colonoscopy; however, as noted above, cost and limited availability of imaging agents and imaging systems currently preclude this. A potentially more realistic intermediate step would be selective use by the screening endoscopist in polyps that are difficult to classify as LGD or HGD/IC on white-light and narrow-band imaging alone, to help decide between referral for local en-bloc resection (ESD/EID) or direct piecemeal resection. Second, fluorescence imaging would ideally be used by the expert endoscopist during local resection itself, to help discriminate between superficial and deep invasion and thereby guide the choice between ESD and EID. In the present study, however, TDR and TBR showed only a non-significant trend towards higher values in deep-invasion lesions, so the feasibility of using fluorescence intensity to predict depth of invasion could not yet be established and warrants further dedicated study. Ultimately, the study should assess the clinical utility and societal benefits of this approach. Another important avenue for research is the evaluation of CEA-targeted endoscopy in response assessment following chemoradiotherapy in patients with locally advanced rectal cancer. Since surgery can be avoided in patients who achieve a complete clinical response, fluorescence-guided flexible endoscopic evaluation using SGM-101 could improve the stratification of patients into complete and incomplete responders, thereby optimizing treatment decisions.

 In this proof-of-concept study, real-time visualization with the CEA-targeted fluorescent agent SGM-101 enabled the identification of HGD/IC foci within otherwise benign polyps, facilitating accurate discrimination between fully benign polyps and those that require en-bloc resection. This highlights another important clinical application of fluorescence-guided endoscopy, broadening the potential of this technology. In vivo validation of flexible fluorescence endoscopy is essential for further advancement. Separately, clinical utility remains to be demonstrated in future, larger prospective trials.

## Supplementary Information

Below is the link to the electronic supplementary material.

Supplementary Material 1 (DOCX 2.08 MB)

## Data Availability

Data are available from the corresponding author upon reasonable request.
