# Supplementary material for: CEA-targeted fluorescence endoscopy for the detection of high-grade dysplasia and early invasive rectal cancer: A proof-of-concept trial
Source: Eur J Nucl Med Mol Imaging. 2026 Sep 3;53(13):8463–77. doi: 10.1007/s00259-026-08155-z (PMC13727823; doi:10.1007/s00259-026-08155-z)
Supplement: Supplementary file 1 [file 259_2026_8155_MOESM1_ESM.docx]

**CEA-targeted fluorescence endoscopy to detect high-grade dysplasia and early invasive rectal cancer: a feasibility trial**

*M.I. Warmerdam^1^, T.H. Dijkhuis^1^, D.J.G. Linders^1^, C.M.W. Pesch^1^, A.S.L.P. Crobach^2^, A. Matoshi^1^, P.J.K. Kuppen^1^, K. Basiliya^3^, S.H. van den Berg^3^, K.C.M.J. Peeters^1^, F.A. Holman^1^, J.S.D. Mieog^1^, A.L. Vahrmeijer^1^,* ***D.E. Hilling^1,4,^ J.J. Boonstra^3^.***

**Authors in bold share last authorship*

1. Department of Surgery, Leiden University Medical Center, Albinusdreef 2, 2333 ZA Leiden, The Netherlands
2. Department of Pathology, Leiden University Medical Center, Albinusdreef 2, 2333 ZA Leiden, The Netherlands
3. Department of Gastroenterology, Leiden University Medical Center, Albinusdreef 2, 2333 ZA Leiden, The Netherlands
4. Department of Surgical Oncology and Gastrointestinal Surgery, Erasmus MC Cancer Institute, University Medical Center Rotterdam, Dr. Molewaterplein 40, 3015 GD Rotterdam, The Netherlands

European Journal of Nuclear Medicine and Molecular Imaging (EJNMMI)

**Corresponding author:**
Drs. M.I. Warmerdam
Albinusdreef 2, 2333ZA Leiden, the Netherlands,
Tel: +31715298420
E-mail: m.i.warmerdam@lumc.nl

**Supplementary table 1**: (Severe) adverse events

| Patient | AE or SAE | Related to study drug or study procedure | Description of event | Outcome |
| --- | --- | --- | --- | --- |
| 3 | AE | Unrelated | Hematoma after traumatic fall on ward one day post-operatively | Recovered |
| 11 | AE | Unrelated | Post-operative increased body temperature | Recovered |
| 12 | AE | Unrelated | Urine retention | Recovered |
| 14 | SAE | Unrelated | Post-operative fever with prolongation of admission | Recovered |
| 14 | AE | Unrelated | Post-operative hypotension | Recovered |
| 17 | AE | Unrelated | Minor post-operative rectal haemorrhage | Recovered |
| 22 | SAE | Unrelated | presacral abscess | Recovered |

**Supplementary table 2:** Patient-level characteristics and diagnostic outcomes

| Patient | Dosage | Pathology | WL Endoscopic assessment | In vivo (quest) | BT (Quest) | BT (PEARL) | TDR (bread loaf) | TBR (bread loaf) | Hypothesis false positive/negative NIRF assessment |
| --- | --- | --- | --- | --- | --- | --- | --- | --- | --- |
| 1 | 10mg | T2 | TP | TP | FN | TP | N.A. | N.A. | FN: Covered T1 by overlying LGD |
| 2 | 10mg | LGD | FP | TN | TN | FP | N.A. | N.A. | FP: Slightly higher CEA at spot of fluorescence |
| 3 | 10mg | T1SM3 | TP | TP | TP | TP | N.A. | 4,3 | N.A. |
| 4 | 5mg | T1SM3 | TP | TP | TP | TP | N.A. | 8,2 | N.A. |
| 5 | 5mg | LGD | TN | TN | TN | FP | N.A. | N.A. | FP: 1) Slightly elevated CEA, 2) Increased tissue thickness, allowing for greater fluorescence signal from deeper layers |
| 6 | 5mg | LGD | TN | FP | TN | FP | N.A. | N.A. | FP: The mildly fluorescent area corresponds to tissue with more advanced LGD, approaching HGD, however, CEA expression is not elevated |
| 7 | 5mg | LGD | TN | TN | TN | FP | N.A. | N.A. | FP: The mildly fluorescent area corresponds to tissue with more advanced LGD, approaching HGD, however, CEA expression is not elevated |
| 8 | 5mg | Irresectable | TP | TP | N.A. | N.A. | N.A. | N.A. | N.A. |
| 9 | 5mg | HGD | FN | TP | TP&FP | TP&FP | 1,7 | 9,1 | This lesion had one TP area (not detected by WL), however there was also a FP hotspot (fluorescent, but LGD) |
| 10 | 5mg | T1SM1 | TP | FN | FN | TP | 1,3 | 3 | FN: Only a slight increase in CEA expression observed between HGD/T1 and LGD (8 vs. 6.3, respectively), accompanied by a low TDR in slices (1.3). Quest appears insufficiently sensitive to detect this difference |
| 11 | 5mg | HGD | TP | TP | TP | TP | 1,2 | 3,5 | N.A. |
| 12 | 5mg | T1SM3 | TP | TP | TP | TP | 1,4 | 2,8 | N.A. |
| 13 | 5mg | LGD | FP | TN | TN | TN | N.A. | N.A. | N.A. |
| 14 | 10mg | T2 | TP | TP | TP | TP | 3,8 | 15,6 | N.A. |
| 15 | 10mg | LGD | TN | TN | TN | TN | N.A. | N.A. | N.A. |
| 16 | 10mg | T1SM3 | TP | TP | TP | TP | 1,5 | 3,4 | N.A. |
| 17 | 10mg | T2 | TP | TP | TP | TP | 2,5 | 3,7 | N.A. |
| 18 | 10mg | T1SM1 | TP | TP | TP | TP | 2,4 | 3,3 | N.A. |
| 19 | 10mg | T1SM1 | TP | TP | TP | TP | 3,2 | 6,7 | N.A. |
| 20 | 10mg | T2 | TP | TP | TP | TP | N.A. | 3,8 | N.A. |
| 21 | 10mg | LGD | TN | TN | TN | TN | N.A. | N.A. | N.A. |
| 22 | 5mg | T2 | TP | TP | TP | TP | N.A. | 3.7 | N.A. |

Abbreviations**:** TP=true positive; FP=false positive; TN=true negative; FN=false negative; N.A.=not applicable; TDR=tumour-to-low-grade-dysplasia ratio; TBR=tumour-to-background ratio; LGD=low-grade dysplasia; HGD=high-grade dysplasia; IC=invasive carcinoma; NIRF=near-infrared fluorescence; CEA=carcinoembryonic antigen; BT=back table whole specimen; WL=white light

**Supplementary figure 1**: Representative full imaging cascade of colorectal polyps from in vivo endoscopy to ex vivo bread loaf analysis.

Three representative polyps, arranged from left to right by increasing pathologic stage (pLGD, pHGD, pT2). From top to down: white-light endoscopy (Olympus GIF-EZ1500), ex-vivo back-table imaging (Quest Spectrum), fluorescence bread loaf images using heatmaps (higher signal = warmer colours), annotations of same bread loaf based on H&E (yellow=mucosa, purple=LGD, green=HGD/IC), and corresponding CEA histopathology. Case 1 shows a fully benign LGD polyp, with corresponding absence of fluorescence and low CEA-staining. Case 2 shows a lateral spreading polyp with a focus of HGD, with corresponding fluorescence and increase in CEA-staining. Case 3 shows a polyp with LGD on the sides with a pT2 component in the centre, with corresponding strong fluorescent signal. The arrow indicates focal hotspot. Abbreviations: WL=white-light; NIRF=near-infrared fluorescence; H&E=haematoxylin–eosin; CEA=carcinoembryonic antigen; LGD=low-grade dysplasia; HGD/IC=high-grade dysplasia/invasive carcinoma; True negative=absence of HGD/IC and no fluorescence; True positive=presence of HGD/IC with a fluorescent signal**.**

**Supplementary figure 2**: TDR, TBR and depth of invasion

TDR and TBR values in superficial versus deep invasion, assessed by back-table and bread-loaf imaging. Superficial invasion includes HGD and T1SM1; deep invasion includes T1SM2–3 and T2. Data shown as mean ±SD with p-values from group comparisons. Abbreviations**:** TDR=tumour-to-low-grade-dysplasia ratio (HGD/IC divided by LGD); TBR=tumour-to-background ratio (HGD/IC divided by mucosa); HGD=high-grade dysplasia; IC=invasive carcinoma; LGD=low-grade dysplasia; SD=standard deviation.

**Supplementary figure 3**: Ex vivo comparison of the Quest-Olympus flexible fiber endoscope versus the Quest-Olympus open system, and Quest Olympus rigid laparoscope

This figure illustrates ex vivo fluorescence imaging of a tumour specimen from patient 22 (pT2) using three different NIRF optical modalities: the Quest open camera system, a rigid laparoscope, and a flexible fibre endoscope, all connected to the Quest Spectrum V3. Each modality presents paired images under normal colour illumination (left column) and near-infrared fluorescence (NIRF) monochromatic imaging (right column). Across all imaging techniques, a clear contrast is observed between the tumour and surrounding mucosa, demonstrated by fluorescence signal (white) localized within the tumour and minimal signal (black) in the adjacent mucosa.
